# 3D Visualization and Volume-Based Quantification of Rice Chalkiness In Vivo by Using High Resolution Micro-CT

**DOI:** 10.1186/s12284-020-00429-w

**Published:** 2020-09-16

**Authors:** Yi Su, Lang-Tao Xiao

**Affiliations:** College of Bioscience and Biotechnology, Hunan Agricultural University, Changsha, China

**Keywords:** Rice chalkiness, 3D visualization, Volume based quantification, Micro-CT, In vivo analysis

## Abstract

**Background:**

Rice quality research attracts attention worldwide. Rice chalkiness is one of the key indexes determining rice kernel quality. The traditional rice chalkiness measurement methods only use milled rice as materials and are mainly based on naked-eye observation or area-based two-dimensional (2D) image analysis and the results could not represent the three-dimensional (3D) characteristics of chalkiness in the rice kernel. These methods are neither in vivo thus are unable to analyze living rice seeds for high throughput screening of rice chalkiness phenotype.

**Results:**

Here, we introduced a novel method for 3D visualization and accurate volume-based quantification of rice chalkiness in vivo by using X-ray microcomputed tomography (micro-CT). This approach not only develops a novel volume-based method to measure the 3D rice chalkiness index, but also provides a high throughput solution for rice chalkiness phenotype analysis by using living rice seeds.

**Conclusions:**

Our method could be a new powerful tool for rice chalkiness measurement, especially for high throughput chalkiness phenotype screening using living rice seeds. This method could be used in chalkiness phenotype identification and screening, and would greatly promote the basic research in rice chalkiness regulation as well as the quality evaluation in rice production practice.

## Background

Rice is the staple food for more than two thirds of the population in China and over half of the population in the world, thus previous research on rice grain yield and quality has attracted attention worldwide (Zeng et al. 2017). Rice chalkiness is one of the key indexes determining both the grain quality (appearance, processing, milling, storing, eating, and cooking quality) and the sales price (Fitzgerald et al. 2009; Siebenmorgen et al. 2013). Rice chalkiness is the opaque part of the endosperm in the rice kernel and it is observed to be in white when comparing to the relatively transparent rest part. According to its location in the kernel, chalkiness traits can be grouped into 3 types of white-belly, white-core and white-base (Yoshioka et al. 2007; Bowles 2012). Previous research showed that rice chalkiness formation was related to abnormal carbohydrate metabolism such as starch biosynthesis and cell wall growth, the opaque part is the location in endosperm with loosely packed storage starch granules (Xi et al. 2014). The chalky appearance is associated with the development of numerous tiny air spaces between loosely packed starch granules and the resulting change in light reflection (Tashiro and Wardlaw 1989). Rice chalkiness is a complicatedly quantitative trait which mainly accumulates at the grain filling stage. Therefore, it is controlled by multiple classes of genes involved in assimilate accumulation in endosperm and is also influenced by multiple environmental factors (Lanning et al. 2011; Bowles 2012; Wada et al. 2019). Although many QTLs controlling rice chalkiness have been reported by various research groups (Li et al. 2014; Qiu et al. 2017; Wang et al. 2018), the underlying molecular mechanism regulating rice chalkiness is far from clear. One of the bottlenecks is the deficiency of accurate and high throughput rice chalkiness quantification methods using living rice seeds to support the highly efficient in vivo screening for mutants in the rice chalkiness phenotype.

Quantitative indexes describing rice chalkiness mainly include chalky rice rate and chalkiness degree. In general, the operational processes in traditional chalkiness measurement methods are usually normalized by some international standards (e.g. Rice-specification. International Standard, ISO 7301:2011) or local standards (e.g. Milled rice. National Standard of the People’s Republic of China, GB/T 1354–2018.) which have played important roles both in commodity inspection and basic researches. Up to date, the traditional methods based on naked-eye observation and artificial regionalization are still widely used in rice chalkiness quantification (Yoshioka et al. 2007; Bowles 2012). Some imaging methods based on digital images and image processing software have been developed to measure and categorize rice chalkiness. Rice chalkiness has been scanned into 2D images based on grayscale value differences between chalky and normal regions in the rice kernel, and the chalky part in the kernel has been finely classified and marked in the image (ISO 7301:2011; Chen et al. 2011; Chen et al. 2013). Algorithm based on support vector machine (SVM) has also been employed to analyze rice chalkiness by using multiple images captured from different angles of milled rice (Sun et al. 2014). Scanning electron microscopy has been usually employed to reveal the density of starch at the μm-scaled level and then indirectly reflect the differences between the chalky and normal regions in the image (Li et al. 2014; Yu et al. 2017). Because of the advances in objectivity and accuracy, these image processing methods have been frequently used as alternative methods to naked-eye observation in rice research and breeding programs.

However, these non-objective and time-consuming traditional methods only use milled rice as materials, usually showing poor consistency and objectivity. Moreover, the broken kernels in glum removing and milling would also decrease the measurement accuracy, and the small sized chalkiness deep inside the rice kernel hardly observable for naked-eyes would also result in reduced chalky rice rate. On the other hand, if a non-chalky part is located behind the chalky part, it would be mistakenly regarded as chalky part by naked-eye and projection-based image analysis software, since these methods are unable to distinguish. Therefore, these methods could hardly meet the urgent needs for the efficient and accurate measurement of rice chalkiness using living rice seeds for the identification of chalkiness related phenotypes.

As a matter of fact, rice chalkiness appears as an amorphous cubic structure in the rice kernel, thus measuring the volume instead of the projection area in rice chalkiness quantification is far more meaningful for both the research and practice. Regretfully, through the current image processing methods, rice chalkiness is all measured in 2D. These images of milled rice are captured by digital camera or scanner from outside. Thus the chalkiness on rice surface is easily detected, but the internal chalkiness related characteristics, are hardly revealed. Scanning electron microscopy which has been employed to analyze the compactness of starch accumulation, could indirectly reflect the rough property of rice chalkiness. However, only a very small area of a rice section can be observed in scanning electron microscopy. Neither the chalky boundary could be well distinguished nor the chalkiness volume be quantified. To be able to accurately analyze internal information about location, shape and volume of rice chalkiness, 3D measurement method for rice chalkiness is urgently needed.

On the other hand, previously reported chalkiness quantification methods only use milled rice as materials, unable to analyze chalkiness in vivo in living rice grains. Since rice chalkiness is located inside the endosperm, processes of glume removing and milling are essential for methods based on naked-eye observation or image scanning. However, these processes are usually accompanied with the embryo destruction, thus the biological activity of the milled rice as a seed would be completely lost. Similarly, scanning electron microscopy method is also destructive, because the process of nanogold coating in sample preparation and high energy electron impact in scanning would seriously reduce biological activity of rice embryo. Therefore, in vivo rice chalkiness quantification method using living rice seeds is also needed by the basic research fields.

X-ray microcomputed tomography (micro-CT) is a nondestructive imaging technique that can be used to generate a series of consecutively cross-section digital images of a physical object with micrometer- and submicrometer-scale resolution (Starosolski et al. 2015). The absorption of X-rays as an index of an object’s physical properties offers the possibility for spatial segmentation based on the matrix X-ray density in biological samples. Through the 3D image reconstructed from these 2D cross-section images, it allows to visualize and quantify X-ray density related biological traits both in 3D and in vivo. Because of its nondestructive characteristics, micro-CT has been previously used to analyze the features of live animal organs/tissues (Liu et al. 2012; Starosolski et al. 2015). Recently, micro-CT has been preliminarily introduced to visualize and quantify morphology characteristics of plant organs/tissues, such as xylem (Brodersen et al. 2011; Knipfer et al. 2015), root (Cuneo et al. 2016), leaf (Kaminuma et al. 2008; Dhondt et al. 2010), flower and grain (Staedler et al. 2018). It has been also used to study the root architecture and interaction with soil microorganisms (Verboven et al. 2012; Mairhofer et al. 2013; Earles et al. 2018). Several researches have paid attention to the analysis of water and starch distribution in the stem of woody plants (Mairhofer et al. 2013). Recently, X-ray computed tomography started to be applied in large-scale assessment of grain and tiller architecture traits in rice (Wu et al. 2019; Hu et al. 2020). Hence, the once difficult 3D measurement of rice chalkiness and its spatial localization could be explored in vivo through the 3D micro-CT technology.

Here, we employed micro-CT scanning and 3D reconstruction techniques to analyze rice chalkiness in living rice seeds. The volume, 3D shape, and location of chalkiness part in the rice kernel were accurately defined. This approach also provided a high throughput solution for rice chalkiness phenotype analysis in vivo and would greatly help the research of rice chalkiness traits.

## Methods

### Sample Collection and Preparation

Several rice (*Oryza sativa* L.) varieties with diverse chalkiness traits, 330 grains of Zhenshan 97B and 30 grains of Xiangzaoxian series (Xiangzaoxian, X226, X220 and X191), were used to perform X-ray scanning in micro-CT. Living rice seeds were collected from the Rice Germplasm Resource Bank of Hunan Province. Brown rice was prepared by removing the husk using a Mini De-husker (Taizhou Cereal Instrument Co. Ltd., Zhejiang, China), and the milled rice was prepared by a mini milling machine (LTJM160, Taizhou Cereal Instrument Co. Ltd., Zhejiang, China).

### Rice Scanning Through Micro-CT

Rice grains were embedded in Super Light Clay (ordered from Alibaba, China). Samples were loaded into SkyScan 1172 Micro-CT (Bruker, Belgium) containing a cone beam X-ray source with < 5-μm focal spot, and a sealed, fully distortion corrected, air-cooled, 10 Mp, 12-bit CCD camera that is fiber-optically coupled to a Gd_2_O_2_S scintillator. The samples were positioned at proper distances from the X-ray source according to the sample size and scanning resolution. The scanning resolution represents the distance between adjacent cross-section images. The micro-CT operation includes resolution setting, current and voltage setting, program running and data saving according to the operation manual. Scanning time depends on sample size and resolution. The sample can be rotated in the sample chamber and scanned in three dimensions. The related parameters employed in X-ray scanning were shown in Table 1. Sample was scanned by X-ray. The signals of transmitted X-ray were acquired according to the set resolution and cross-sections were imaged by an X-ray camera. The scanning results were preferred to be exported as DCM format files comparing to other supported formats (e.g., BMP, Tiff, JPG), since DCM files containing 3D information are more compatible with the subsequent 3D reconstruction software.

**Table 1:** Related parameters employed in X-ray scanning

Resolution	15 μm	13.5 μm	10 μm	5 μm	2.5 μm
Voltage (kV)	50	50	50	50	50
Electric current (μA)	150	150	120	140	140
Scan time (min)	11	27	15	15	35
Size of data storage (G)	1.5	11.8	6.8	10.3	32.9

### Separation and 3D Reconstruction of Chalkiness

DCM files were imported into Mimics Innovation Suite (MIS) trial version (Materialise, Belgium) by using import method “Strict DICOM 3.0” (digital imaging and communications in medicine 3.0). A series of monochrome cross-section images was exhibited in three views of top-bottom, right-left and front-back. Separation/segment of rice chalkiness and 3D reconstruction were carried out through the software integration tools in MIS, including “Thresholding”, “Contrast”, “Region Growing”, “Edit Masks”, “Morphology Operation” and “Calculate 3D”. The values in “Threshold” were adjusted to select the whole solid area which displayed in colored mask (M1). “Region Growing” was employed to separate the whole rice area in M1 from background and support material, and to display the separated rice in a new mask (M2). In order to separate rice chalkiness which locates in dark area, the parameter ranges in “Threshold” and “Contrast” were adjusted and the dark area was selected and highlighted in a new mask (M3). The background was removed by using “Region Growing” and “Edit Masks”. “Morphology Operation” was employed to dilate the dot array and filled the holes. The separated chalkiness exhibited in mask (M4). “Measurements” was applied in measuring the distance between two points and area of selected region. By using “Calculate 3D”, the cross-section in M2 and M4 were reconstructed as 3D objects which respectively represented cubic rice and chalkiness. The reconstructed 3D objects were showed in a window and allowed free rotation and viewing. The accurate volume of rice kernel and chalkiness were directly readable in “3D Properties” (Supplemental video 1). The percentage of rice chalkiness was then calculated based on the volume data for both the rice kernel and chalkiness.

### Software and Computer Hardware

MIS and Chalkiness 2.0 were running in a desktop computer powered by 64 bit Windows 10 (Microsoft, USA). To favorably perform 3D reconstruction of rice grains, optimal hardware configuration included an Intel Core i7-9750H CPU (64 bit), 32 gigabytes of memory and a GeForce RTX 2060 graphics card (NVIDIA, USA).

### 2D Chalkiness Area Analysis by Using Chalkiness 2.0

In our previous research, Chalkiness 2.0 software was developed to quantify the rice kernel and chalkiness sizes based on 2D image area analysis (Chen et al. 2011). A free evaluation version can be downloaded at http://hnspb.cn/chalkiness2.0/. In this study, Chalkiness 2.0 was employed as the control and supplemental method for the rice chalkiness and size analysis. The milled rice grains (≥ 30) were spaced on dark plane and an image was captured by a scanner or camera. The supported format image (JPG, BMP and PNG) was imported into Chalkiness 2.0 software. The kernel profiles were auto-recognized with an encircled red line, and the chalkiness area was separated with white line. The chalkiness related parameters (including grain number, relative chalkiness area, chalkiness degree and chalky rice rate) and kernel size were displayed in a new window by using “Calculate”. The test report was eventually generated in an Excel format file by using “Printing report”.

### Seed Germination and Growth Condition

The rice seeds were soaked in Petri dish (9 cm diameter) with two sheets of filter paper and 20 mL sterile water. Petri dish was placed in a constant temperature incubator for 24 h at 30 °C. Water was removed and the seeds were washed 3 times with sterile water. Then Petri dish was placed in a constant temperature incubator for 12 h in dark at 30 °C. Seed germination was observed afterwards. In addition, the germinated seeds were planted in 5 kg soil with 1 L of Kimura B solution (Ma and Takahashi 1990) and cultured in green house in 16 h light/ 8 h dark at 30 °C. The growth status was observed after 7 days.

## Results and Discussion

### Principles and Processes of Rice Chalkiness Quantification

**Fig. 1:**
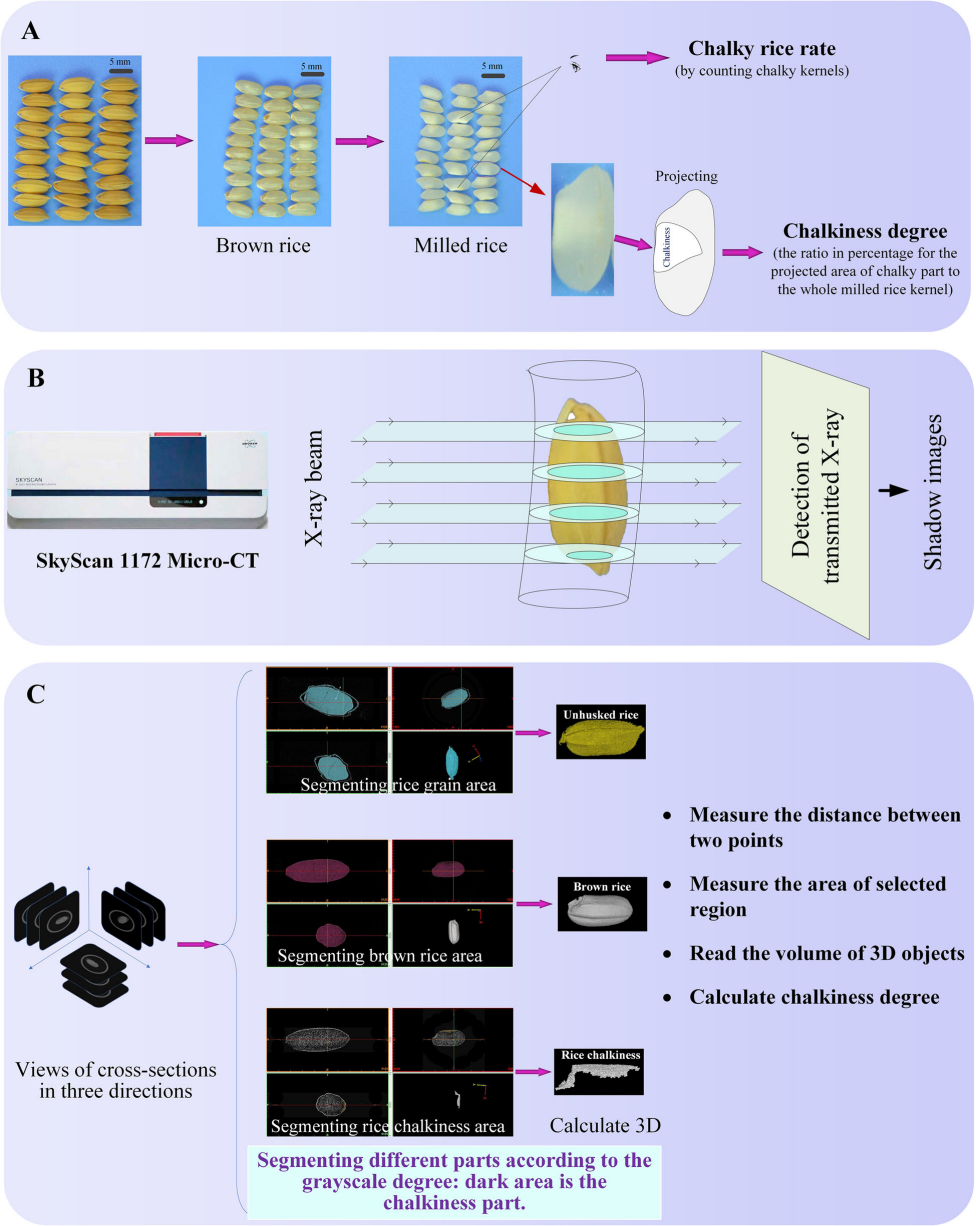
Principles and processes of rice chalkiness quantification. **a** Principles and processes of rice chalkiness quantification in traditional methods. Chalkiness analysis includes glume removing and milling, following by naked-eye observing and then the area-based chalky rice rate calculation. The chalkiness degree was evaluated by the projected area rate between chalkiness area and milled rice area in an image processing software. **b** Principles of X-ray ray scanning by micro-CT system. SkyScan 1172 Micro-CT was employed to scan living rice seeds or milled rice. **c** Principles of chalkiness separation/segmentation. Chalkiness part was separated according to the grayscale degree. After 3D reconstruction, the cubic parameters were analyzed

The traditional methods for rice chalkiness evaluation includes the processes of glume removing and milling, followed by naked-eye observing to count the chalky kernel percentage and judge the chalky rice rate (Fig. 1a). The chalkiness degree is represented by the ratio in percentage for the respective projected areas of chalky part to the whole milled rice kernel (Fig. 1a). In practice, the image of milled rice grains is captured by a scanner or camera. The image processing software is employed to separate the rice chalky part according to the different densities, and calculate the relative area of chalkiness (Chen et al. 2013). In our previous research, we developed a Chalkiness 2.0 software which could carry out high-throughput analysis of rice chalky parameters, including the grain number, relative chalkiness area, chalkiness degree, chalky rice rate, and rice length and width (Chen et al. 2011). Regretfully, those 2D area based traditional methods are unable to quantify the chalkiness volume and analyze the inner structure of 3D shaped chalky part.

In this study, we employed micro-CT and 3D reconstruction to visualize and quantify the chalkiness in the rice. The basic principle of microtomography was shown in Fig. 1b. In the simplest case, it is described as the scanning with a parallel X-ray beam. Any X-ray shadow image is corresponding to a two-dimensional projection from the rice. In this approximation, each point on the shadow image contains the integration of absorption information inside the rice seed in the corresponding partial X-ray beam. When using micro-CT machine, the sample can be rotated in the sample chamber. Thus rice sample can be scanned in three dimensions and the captured cross-section images revealed the 3D information. In parallel geometry, one can divide the problem of a three-dimensional reconstruction from two-dimensional projection to the serial reconstruction of two-dimensional object slices from one-dimensional shadow lines. In our proposed method for 3D visualization and quantification of rice chalkiness, not only milled rice but also the living rice seeds could be directly used to perform computed tomography in micro-CT system (Fig. 1b).

Then after, a series of cross-section images captured by micro-CT system were used for the chalkiness quantification according to the principles of segmentation and 3D reconstruction in MIS (Fig. 1c). All the cross-section images were imported into MIS. The outlines of rice grains were displayed as grayscale images in three windows which represented the three dimensions. Since the grayscale degree was negatively correlated with the density, the chalkiness part appeared in the dark area was easily distinguishable from the non-chalkiness part. By using a series of segment tools, the rice grain, chalkiness and non-chalkiness parts were auto-separated and highlighted with different colors. 3D images of the intact grain, the chalkiness and non-chalkiness parts in the kernel could be respectively reconstructed through “Calculate 3D” tool (Fig. 1c, Supplemental videos 1 and 2). The volumes of the 3D objects were directly provided by the built-in software while other related parameters were manually calculated.

Comparing to the traditional methods, 3D visualization based on micro-CT can easily and accurately define the volume, 3D shape, and location of chalkiness part in the rice kernel. The volumes of chalkiness part and brown rice were simultaneously calculated by MIS and then chalkiness degree was precisely quantified.

### Microtomography Analysis of Rice in Different Resolutions

**Fig. 2:**
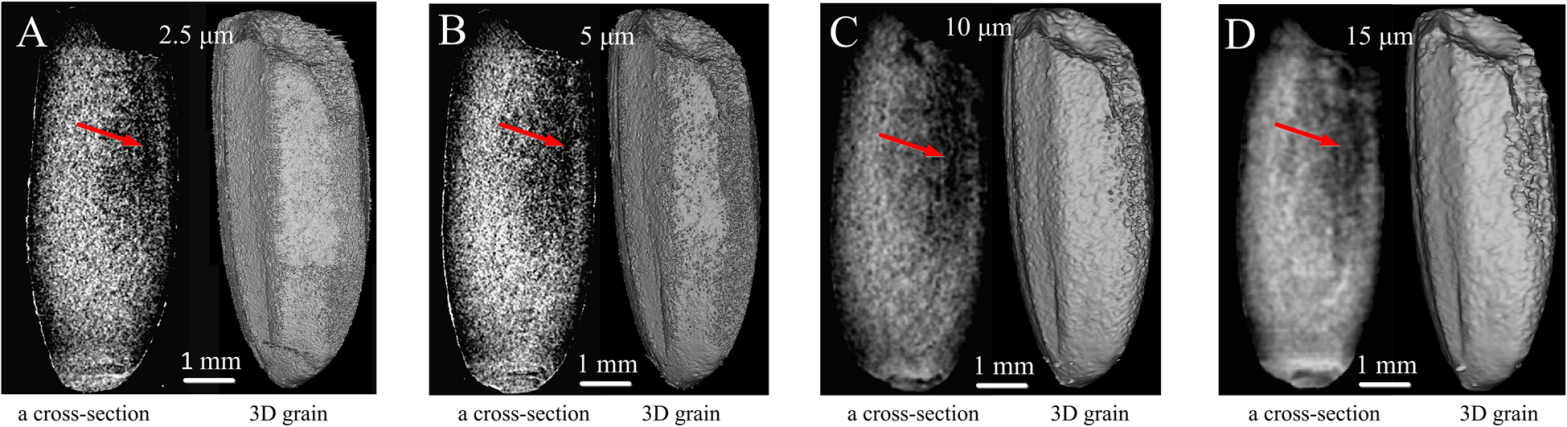
3D reconstruction of rice (X220) under different X-ray scanning resolutions. A cross-section image and reconstructed 3D milled rice kernel with whit-belly chalkiness under X-ray scanning with resolution of 2.5 μm (**a**), 5 μm (**b**), 10 μm (**c**) and 15 μm (**d**). Dark areas indicated by red arrow represented the location of rice chalkiness

We compared the quality of the cross-section images and reconstructed 3D images under different scanning resolutions ranging from 2.5 μm to 15 μm. The chalkiness could be well revealed under 2.5 μm and 5 μm resolutions (Fig. 2a and b). Under 10 μm and 15 μm resolutions, the cross-section images were a little vague and the reconstructed rice grain image showed relatively low accuracy, but the chalkiness areas still could be roughly distinguished (Fig. 2c and d). Generally, the higher the resolution was employed, the longer the scanning time was needed. Under 2.5 μm resolution, it took over 30 min per test and generated over 30 gigabytes data per grain, which is not convenient for subsequent computerized data processing. Scanning under the resolutions range from 5 μm to 10 μm showed relatively high resolution with less time consumption (about 15 min per test) and proper data size (about 10 gigabytes per grain). Therefore, resolutions ranging from 5 μm to 10 μm are suggested for high resolution analysis of rice chalkiness, while resolutions ranging from 10 μm to 15 μm are suitable for rough quantification and high throughput analysis of rice chalkiness due to the significantly decreased scanning time. In addition, the current and voltage parameters depend on the sample properties. The different resolutions corresponded to the different settings of voltage and current. Higher voltage and current represent higher X-ray power and stronger transmission capability. The rice seed showed a low hardness and density. Thus the voltage and current were set in a low value, and the average transmission capability of X-ray maintained in the range of 40%–60% according to the sample size and embedding matrix. Too low or too high power will result in the loss of image details. The subsequent segmentation of chalkiness will be more difficult, and the resolution of 3D reconstruction will be also reduced.

### 3D Chalkiness Analysis of Different Chalkiness Types

**Fig. 3:**
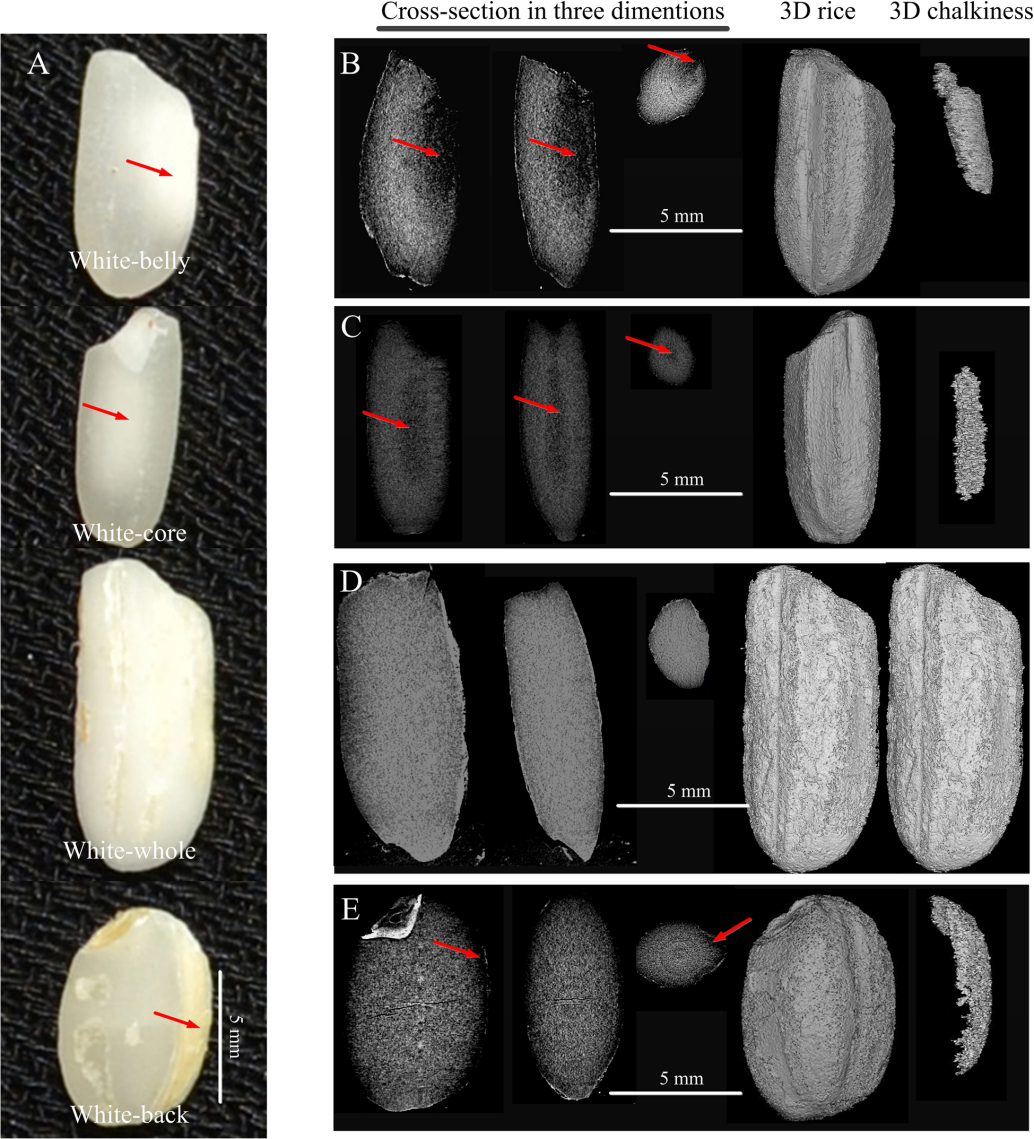
The shape and location of rice chalkiness. **a** milled rice with chalkiness of white-belly (X220), white-core (X226), white-whole (Xiangzaoxian) and white-back (X191) respectively; Cross-section images, reconstructed 3D rice images and reconstructed 3D chalkiness images of milled rice with chalkiness of white-belly (**b**), white-core (**c**), white-whole (**d**) and white-back (**e**) respectively. Dark areas indicated by red arrows represent the location of rice chalkiness

In this study, we collected samples with 4 types of rice chalkiness, i.e. white-belly, white-core, white-whole and white-back, from different cultivated varieties for the volume-based 3D chalkiness quantification (Fig. 3a). Milled rice was scanned by X-ray under 5 μm resolution. The chalkiness areas and their borders were easily observed in cross-sections (Fig. 3b-e). The chalkiness areas in cross-sections were selected and reconstructed through the MIS (Fig. 3b-e). The spatial location and shape of cubic chalkiness part were visualized through the reconstructed 3D chalkiness image, and the chalkiness degree was calculated based on the volume data provided by the built-in software (Table 2). In previous research, we developed a projection-based software (named Chalkiness 2.0) to evaluate rice chalkiness (Chen et al. 2011). In this study, the chalkiness degree in different chalkiness type rice was also detected by Chalkiness 2.0 as the traditional 2D method. The results indicated that the values of chalkiness degree detected by 2D method were distortedly higher than that by 3D method in white-belly, white-core and white-back types of rice. Due to the clarification of the non-chalky part behind the chalky part in 3D method, which is usually regarded as chalky part in 2D method, the 3D method has obvious advantages over the 2D method in the accuracy to detect real chalky part. Moreover, the values calculated in MIS showed the veritable object volume because the cross-section images captured by micro-CT accurately reserve the native size of sample (Table 2), but the area values evaluated in the 2D method were usually relative.

**Table 2:** Chalkiness quantification of rice kernel samples with different chalkiness types

samples	VG (mm^3^)	VC (mm^3^)	CD-3D (%)	CD-2D (%)	Significance between 3D and 2D methods
white-belly	9.96	0.54	5.4	18.8	**
white-core	7.91	0.42	5.3	15.6	**
white-whole	13.65	13.65	100	98.7	NS
white-back	14.06	0.79	5.6	9.9	**

Note: VG (mm^3^) and VC (mm^3^) respectively represented the volume of grain and chalkiness detected by 3D method. CD-3D (%) and CD-2D (%) respectively represented the chalkiness degree detected by 3D method and 2D method. ** represented that the difference was extremely significant. NS represented “not significant”

### High Throughput Analysis of Rice Chalkiness

**Fig. 4:**
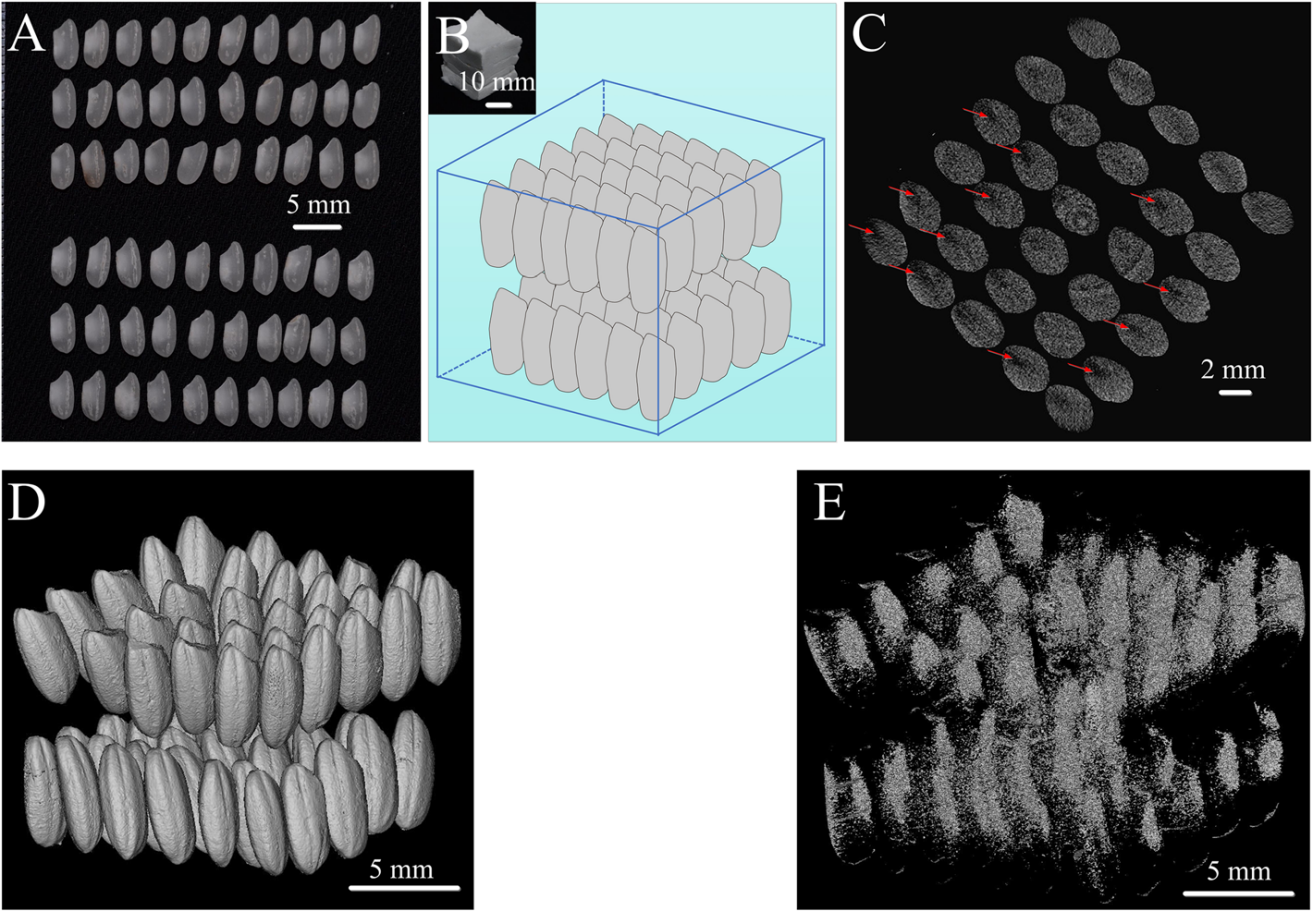
High-throughput analysis of 3D rice chalkiness by using micro-CT scan and 3D reconstruction. **a** Milled rice (Zhenshan 97B); **b** Milled rice were embedded in Super Light Clay (about 2 cm × 2 cm × 2 cm); **c** One cross-section of milled rice grains under 13.5 μm resolution, and the red arrows represent the location of rice chalkiness; **d** Separated and reconstructed 3D image of milled rice embedded in Super Light Clay; **e** Separated and reconstructed 3D image of chalkiness. Dark areas indicated by red arrows represent the location of rice chalkiness

According to the international standards (e.g. ISO 7301:2011) or local standards in China (e.g. GB/T 1354–2018), samples for a single test generally require more than 30 rice grains, thus high-throughput analysis of chalkiness is very important. We tested the maximum detectable number of rice grains under different resolutions by one X-ray scan of the micro-CT system. Under resolution lower than 5 μm, multiple rice grains can be completely scanned (Table 3). Resolutions ranging from 10 μm to 15 μm can be applied in high-throughput analysis of rice chalkiness since the chalkiness can be well visualized through micro-CT (Table 3, Fig. 4). In order to conveniently distinguish the grain area borders, we employed Super Light Clay as the supporting substance because its density is far below the rice kernel density and can be easily segmented from cross-sections by using the MIS. Through micro-CT and reconstruction, the cubic rice chalkiness parts in as many as 60 rice grains can be well located, visualized and their volume data can be also accurately calculated at the same time. The chalky rice rate can be also calculated through a series of cross-section images (Fig. 4).

**Table 3:** Resolution of micro-CT and the detectable number of rice grains

Resolution	13.5 μm	10 μm	5 μm	2.5 μm
Detectable number	40–60	20–30	5–10	1
Sample holder	Custom	Custom	Glass tube	Glass tube

### In Vivo Analysis of Rice Chalkiness

**Fig. 5:**
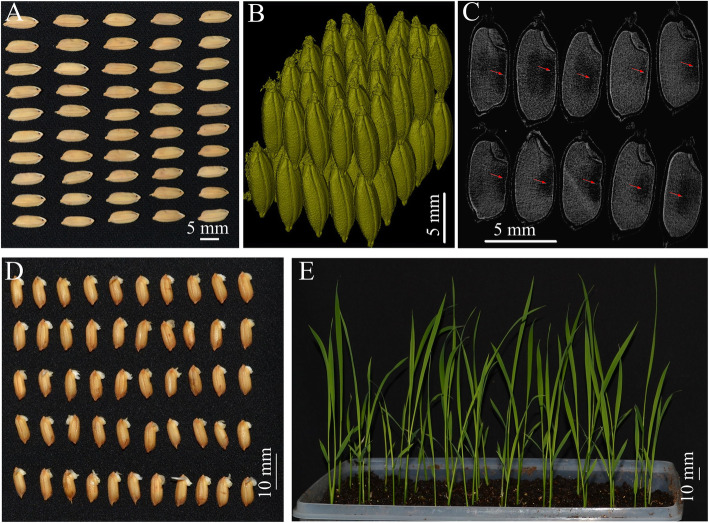
in vivo analysis of rice (Zhenshan 97B) chalkiness. **a** 50 living mature rice seeds; **b** Separated and reconstructed 3D image of rice grain; **c** One of cross-sections of rice grain; **d** Germination analysis of rice grain after X-ray scanning. **e** Growth analysis of rice seedlings after X-ray scanning. Dark areas indicated by red arrows represent the location of rice chalkiness. Three independent biological replicates were analyzed

Micro-CT system was previously introduced to investigate the plant tissue structures (Kaminuma et al., 2010; Dhondt et al. 2010; Brodersen et al. 2011; Knipfer et al. 2015; Cuneo et al. 2016; Staedler et al. 2018) and the interactions between plant roots and soil (Verboven et al. 2012; Mairhofer et al. 2013; Earles et al. 2018). Generally, these samples have been separated from the living plants. Impressively, we found that living rice seeds can be used for microtomography analysis in this study. To confirm the potential effects of X-ray on the rice seed activity, we monitored the seed germination rate and the seedling growth after the rice seeds were scanned by X-ray under the 13.5 μm resolution. The results of three repeated scans indicated that all rice seeds scanned by X-ray in micro-CT could normally germinate, showing no germination and growth defects (Fig. 5a-e, Supplemental Figure 1). High dose of X-ray and long-time exposure in X-ray would inhibit seed germination and plant growth, since it has been reported that seed germination and plant growth were influenced by exposure to X-rays, in both cases of high and low X-ray doses (Johnson 1936; Genter and Brown 1941; Al-Khayri et al. 2013). At present, CT and micro-CT systems have been widely applied in in clinical treatment since their safety for humans and animals is guaranteed. Previous findings showed that the X-ray doses in CT and micro-CT systems did not affect the plant growth (Zappala et al. 2013). In this study, we also found that the micro-CT scanned rice seeds germinated normally, and the seedling growth showed no negative effects. The current and voltage parameters determine the X-ray dose (power), which depends on the sample properties (e.g. hardness and density). Comparing to the research of Zappala et al. (2013), lower current and voltage, single X-ray exposure, and shorter exposure time were employed in our tests. Moreover, the rice seeds have more protective structures to X-ray exposure comparing to delicate rice roots. Thus the suffering of rice seeds under micro-CT X-ray doses was minimal in this study. Therefore, micro-CT can be used for in vivo and non-destructive analysis of the rice chalkiness, showing the unique advantage over all traditional destructive methods only using milled rice kernels.

### Auto-Calculation of Chalky Rice Rate and Rice Size by Combining with Chalkiness 2.0

**Fig. 6:**
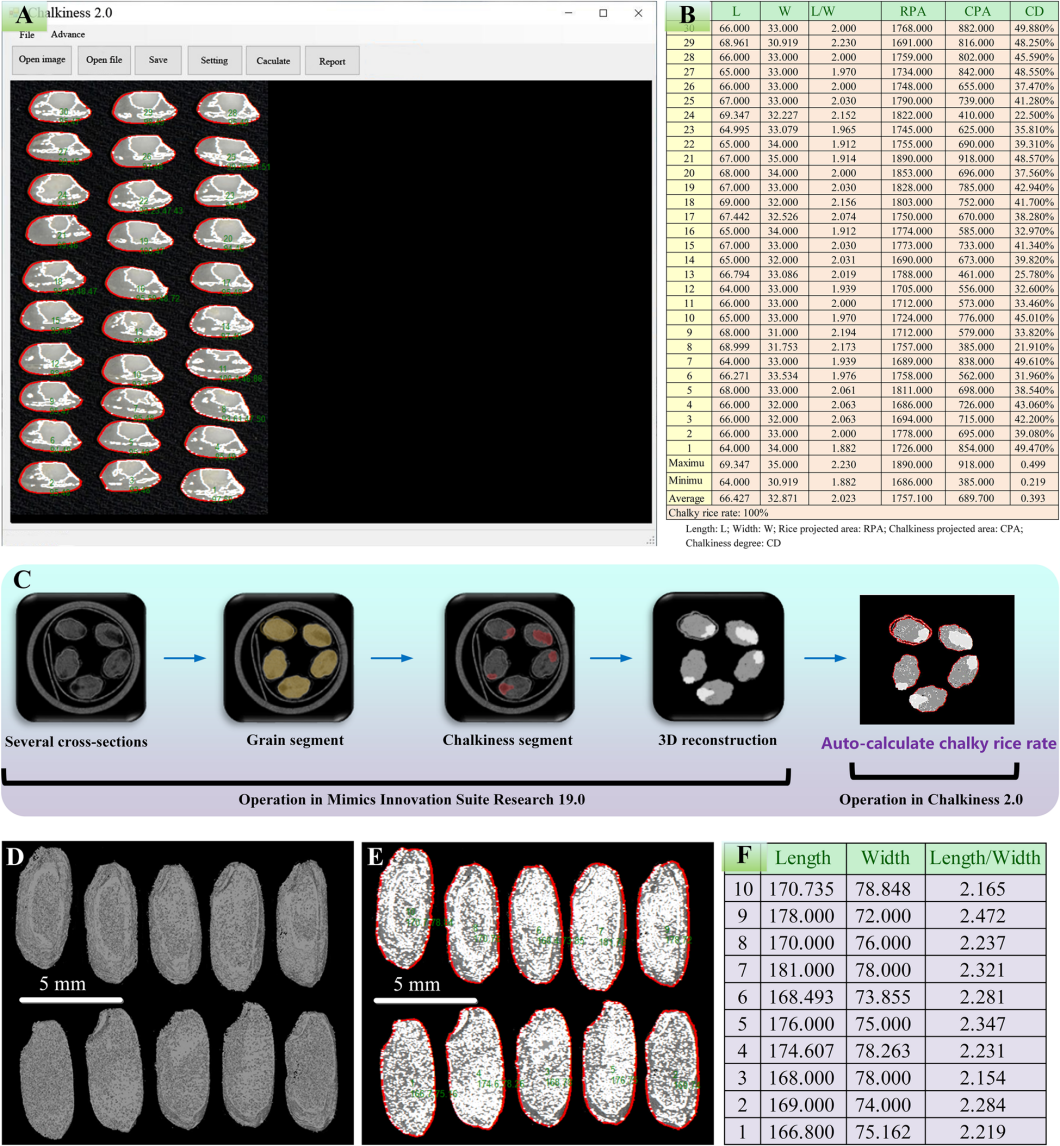
Auto-calculation of chalky rice rate and rice grain size by using micro-CT 3D reconstruction coupled with Chalkiness 2.0 software. **a** Scanned image of milled rice (Zhenshan 97B) imported in Chalkiness 2.0 software, and the rice grain and chalkiness were auto-regionalized; **b** The chalkiness related parameters were auto-calculated, and the test report was exported into an Excel format file; **c** Graphical overview of auto-calculating chalky rice rate based on micro-CT 3D reconstruction coupled with Chalkiness 2.0 software. **d** 20 cross-sections near the maximum section were reconstructed and a plane image was generated; **e** The plane image was import to Chalkiness 2.0 for grain size evaluation; **f** The quantitative results were exported into an Excel file. The values represent the relative length and width

Through micro-CT and 3D reconstruction, the 3D rice chalkiness part was precisely located and visualized, and its volume was accurately quantified (Figs. 4 and 5). The chalky rice rate could be calculated by counting the number of the cross-section images with chalkiness. The rice grain size (length and width) also could be manually measured though the “Measurements” tool in MIS. However, the micro-CT built-in software is limited in auto-calculating the grain size and chalky rice rate. Chalkiness 2.0 was previously developed by the authors’ group (Chen et al. 2011) for high-throughput chalkiness quantification in milled rice according to the grayscale analysis of 2D image captured by a camera or scanner (Fig. 6a and b). According to the principles of micro-CT, a series of 2D images of cross-section layers could be captured and exported as general format images, such as BMP, JPG and TIFF. We tried to directly import these cross-section images into Chalkiness 2.0 software to perform automatic chalkiness analysis. Regretfully, the rice grain and chalkiness in these cross-section images generated by micro-CT were not accurately recognized and regionalized by Chalkiness 2.0.

In order to make Chalkiness 2.0 suitable for auto-calculation of chalky rice rate for micro-CT cross-section images, we further processed the cross-section images. Several adjacent cross-section images in central location were selected and enhanced the contrast in order to facilitate the visualization of the chalkiness region and non-chalkiness region. Through segment tools in MIS, the rice grain and chalkiness were segregated and visualized with different colors. After 3D reconstruction, the cross-section images became smooth plane. Subsequently, the smooth plane image would be well distinguished, and the chalky rice rate would be accurately auto-calculate by Chalkiness 2.0 (Fig. 6c). In most cases, the auto-calculation system (e.g. Chalkiness 2.0) and traditional method (e.g. naked-eye observation) can provide similar chalky rice rate. However, these 3D reconstruction processes are very helpful for founding the small chalkiness part hided in the center of rice.

Similar cross-section image processing can also be used for auto-calculation of rice grain size. Length/width ratio represents the axial maximum length to the radial maximum width. When using Super Light Clay to embed multiple rice grains, it is hard to guarantee that the central axis of all rice grains is in the same plane. To obtain the maximum grain length and width, we designated 10–20 cross-section images near the maximum section and performed reconstruction (Fig. 6a and e). The plane image was imported into Chalkiness 2.0. The length, width and length/width ratio were auto-calculated and the results were exported into an Excel format file (Fig. 6f). Therefore, 3D reconstruction coupled with Chalkiness 2.0 was an ideal solution for auto-calculation of rice size and chalky rice rate. Besides, more cubic information (e.g. size and shape of embryo and endosperm, and the traits of rice glume) could be well visualized and quantified in further researches, which will benefit rice functional genomics and grain quality study. This method also can be applied to the phenotype identification of 3D chalkiness (or some other traits) in QTL and GWAS analysis.

## Conclusion

The traditional rice chalkiness measurement methods are mainly based on naked-eye observation or area-based 2D image analysis and using the milled rice kernel as the materials, thus could not reflect the cubic characteristics of chalkiness in the rice kernel or analyze the chalkiness trait in vivo in living rice seeds. Micro-CT was a powerful tool to visualize the inner structure of bio-samples in vivo with micrometer-scale resolution by using X-ray scan and reconstruction techniques. Through 3D reconstruction and volume calculation, we accurately obtained the information about the volume, shape and localization of chalkiness in living rice seeds. Moreover, micro-CT allows to scan as many as 60 rice grains at once, and the cubic-shaped chalkiness can be visually located in the 3D rice grains. This volume-based and high throughput quantification of rice chalkiness in vivo is a new method to accurately localize and measure the cubic-shaped chalkiness part using the rice grains or even living rice seeds. Our protocol also showed great potential to be applied in chalkiness phenotype screening, quality inspection and non-destructive analysis for other X-ray density related traits. In short, with the unique advantage of using living rice seeds as samples, this in vivo 3D visualization and volume-based quantification of rice chalkiness based on high resolution micro-CT, which is a significant improvement to the traditional naked-eye observation methods and area-based 2D imaging methods, would greatly facilitate the volume based chalkiness quantification in basic research programs, especially the high throughput screening of chalkiness phenotype in large scale QTL and GWAS analysis.

## Additional Files

**Additional file 1.**

**Additional file 2.**

**Additional file 3: Supplemental Figure 1.** Growth analysis of rice seedlings after X-ray scanning.

## Data Availability

All data supporting the conclusions of this article are provided within the article and its supplementary files.
