# Supplementary material for: 3D Visualization and Volume-Based Quantification of Rice Chalkiness In Vivo by Using High Resolution Micro-CT
Source: Rice (N Y). 2020 Sep 16;13:69. doi: 10.1186/s12284-020-00429-w (PMC7494727; doi:10.1186/s12284-020-00429-w)
Supplement: Supplementary file 3 — Additional file 3: Supplemental Figure 1. Growth analysis of rice seedlings after X-ray scanning. [file 12284_2020_429_MOESM3_ESM.docx]

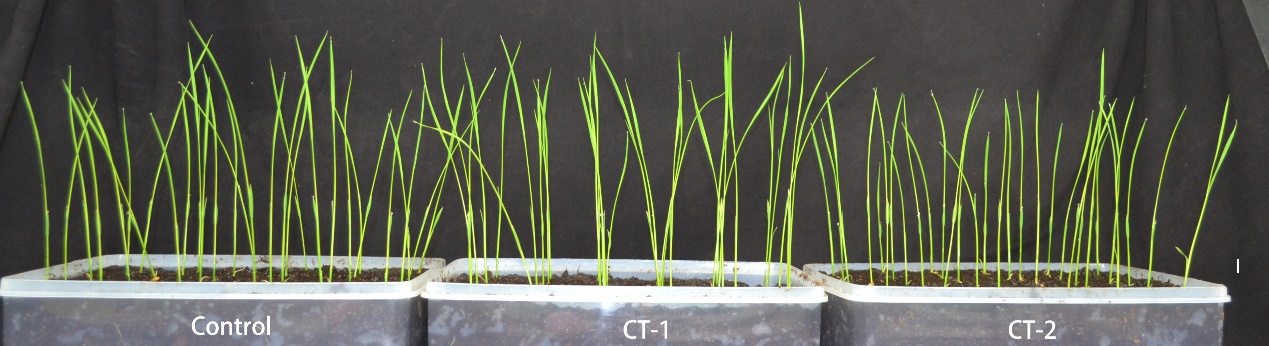


Supplemental Figure 1 Growth analysis of rice seedlings after X-ray scanning. CT-1 and CT-2 represented the X-ray scanned sample.
